# Blockade of CD47 enhances the antitumor effect of macrophages in renal cell carcinoma through trogocytosis

**DOI:** 10.1038/s41598-022-16766-3

**Published:** 2022-07-22

**Authors:** Ha-Ram Park, Seong-Eun Kim, Bhumsuk Keam, Hyewon Chung, Seung Hyeok Seok, Soyeon Kim, Miso Kim, Tae Min Kim, Junsang Doh, Dong-Wan Kim, Dae Seog Heo

**Affiliations:** 1 Seoul National University Cancer Research Institute, Seoul, Republic of Korea; 2 Department of Mechanical Engineering, Pohang University of Science and Technology, Pohang, Republic of Korea; 3 Department of Internal Medicine, Seoul National University Hospital, Seoul, Republic of Korea; 4 Department of Microbiology and Immunology, Seoul National University College of Medicine, Seoul, Republic of Korea; 5 Integrated Major in Innovate Medical Science, Seoul National University Graduate School, Seoul, Republic of Korea; 6 Department of Materials Science and Engineering, Seoul National University, Seoul, Republic of Korea

**Keywords:** Cancer therapy, Renal cancer

## Abstract

Immune checkpoint inhibitors and vascular endothelial growth factor receptor tyrosine kinase inhibitors (VEGFR TKIs) are mainstream treatments for renal cell carcinoma (RCC). Both T cells and macrophages infiltrate the tumor microenvironment of RCC. CD47, an immune checkpoint of macrophages, transmits the “don’t eat me” signal to macrophages. We propose a novel therapeutic strategy that activates the antitumor effect of macrophages. We found that CD47 was expressed in patients with RCC, and high CD47 expression was indicative of worse overall survival in datasets from The Cancer Genome Atlas. We observed that CD47-blocking antibodies enhanced the antitumor effect of macrophages against human RCC cell lines. Trogocytosis, rather than phagocytosis, occurred and was promoted by increased cell-to-cell contact between macrophages and RCC cells. Trogocytosis induced by CD47 blockade occurred in the presence of CD11b integrin signaling in macrophages and was augmented when RCC cells were exposed to VEGFR TKIs, except for sunitinib. In conclusion, this study presents evidence that anti-CD47 blocking antibodies improve the antitumor effect of macrophages in RCC. In combination with VEGFR TKIs, CD47 blockade is a potential therapeutic strategy for patients with RCC.

## Introduction

Renal cell carcinoma (RCC) is the most common subtype of kidney cancer with increasing incidence every year^1^. Recent cancer genome studies have shown that RCC has high intratumoral and intertumoral heterogeneity, making it difficult to establish specific targeted therapeutic strategies and predict their prognosis^2^.

The majority of RCCs are accompanied by Von Hippel-Lindau loss or silent mutations that induce a pseudohypoxia state of tumor cells through hypoxia-inducible factor (HIF) protein accumulation^3^. Several genes involved in tumor proliferation, migration, and angiogenesis, including vascular endothelial growth factor A (VEGF-A), are transcriptionally increased in tumor cells^4^. Vascular endothelial growth factor tyrosine kinase inhibitors (VEGFR TKIs) have been commonly used for the treatment of advanced RCC and have yielded a better response over the years due to continual development^5,6^. Nevertheless, these therapies are still limited in that patients treated with them eventually develop acquired resistance^7,8^.

To overcome the therapeutic limitation of conventional treatment, immune checkpoint inhibitors (ICIs) have been used for RCC^9,10^. Compared with VEGFR TKI monotherapy, VEGFR TKI plus ICI combination therapy has improved the survival of patients with RCC^11–14^. For instance, axitinib plus pembrolizumab showed survival benefits compared with the standard therapy of sunitinib (median progression-free survival, 15.1 months vs. 11.1 months)^15^. However, despite continuous efforts to improve treatment strategies, the prognosis of patients with RCC remains poor^8^. Therefore, novel treatment strategies after ICI failure are needed.

RCC is considered an immunogenic cancer with a high rate of immune cell infiltration in the tumor microenvironment (TME)^16,17^. Macrophages are abundant in the TME of RCC and have been reported to be associated with poor prognosis^18–20^. Tumor-associated macrophages in the TME can play a dual role in either supporting or suppressing tumor cells^21^; thus, their exploitation could lead to effective tumor removal. Among the several immune checkpoints of macrophages, we focused on CD47. CD47 is ubiquitously expressed across various cell types and upregulated in tumor cells compared to normal cells in most cancer types^22–24^. CD47 works not only as a receptor for thrombospondin-1 (TSP-1) but also as a ligand for the signaling regulatory protein alpha (SIRPα)^25^. TSP-1 effects on tumors are multifaceted by regulating various cell functions, including tumor proliferation, apoptosis, cell adhesion, and immune cell activity^26,27^. The increase of TSP-1 brought a reverse effect in the tumor cell progression. For instance, increased TSP-1 and CD47 interaction enhanced tumor progression in T-cell lymphoma and breast cancer, but induced drug resistance in thyroid cancer^27^. Meanwhile, SIRPα ligation by CD47 transmits the “don’t eat me” signal to macrophages, thereby inhibiting phagocytosis^28^. In addition, previous studies have demonstrated that the phagocytic activity of macrophages against tumor cells can be boosted through blocking the CD47-SIRPα interaction^29–32^. Increased phagocytosis was observed regardless of macrophage subtypes, such as M1, M2, or tumor-associated macrophages, as a result of CD47 blockade^29^. Various anti-CD47 blocking antibodies have been developed and have shown favorable responses in hematologic malignancies and a few solid tumors^33,34^.

Our study explored the effect of CD47 blockade on the antitumor effects of macrophages against RCC cell lines. We analyzed the correlation between CD47 expression and patient survival and evaluated the antitumor effect of macrophages against CD47-expressing RCC cells when CD47-blocking antibodies interrupted the interaction between CD47 and SIRPα.

## Results

### CD47 expression in renal cell carcinoma

**Figure 1:**
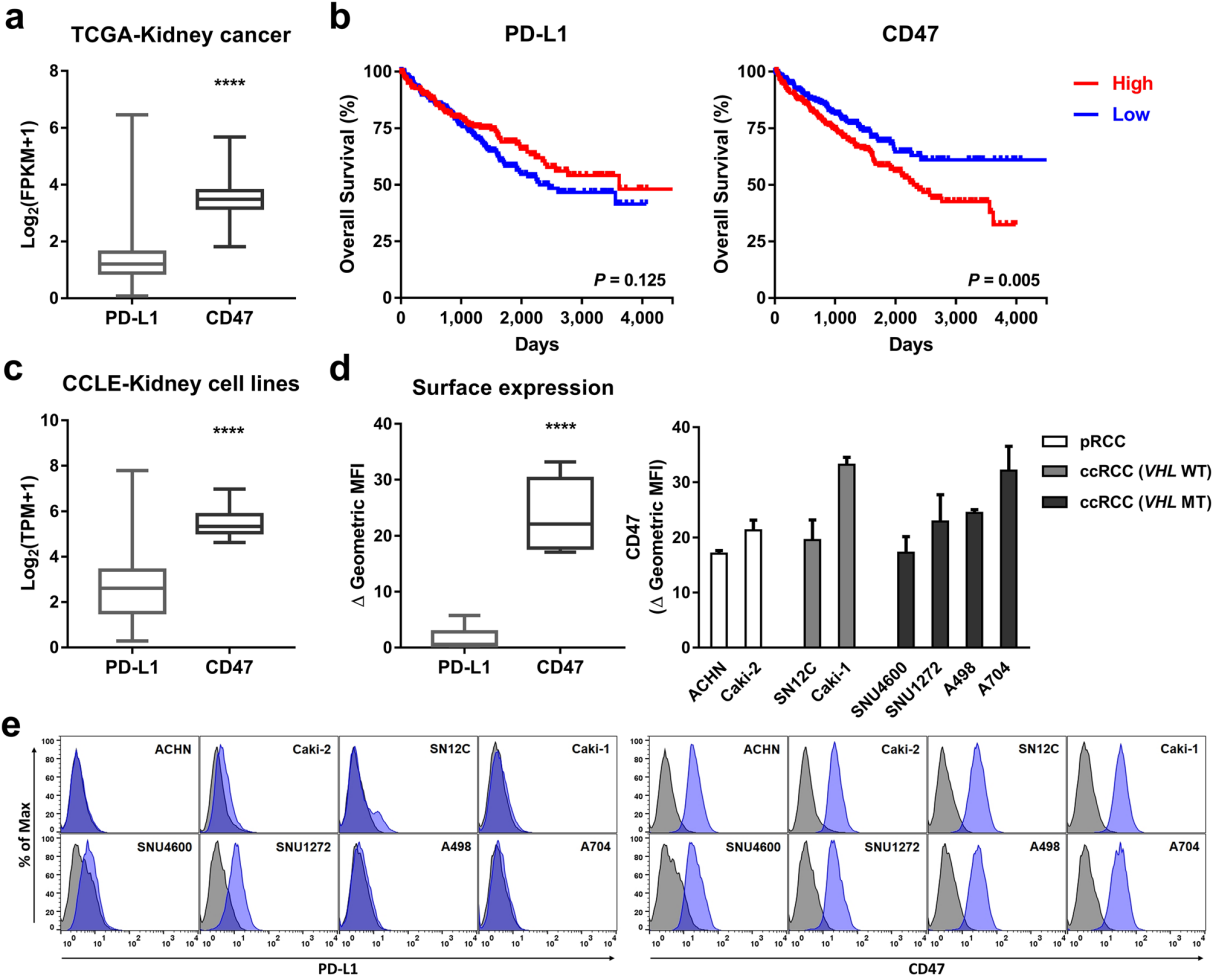
CD47 is highly expressed in renal cell carcinoma (RCC) and is associated with a worse prognosis. (**a**) A comparison of PD-L1 and CD47 expression levels in patients with RCC (n = 1,017) using TCGA data. The box and whisker plot shows the median, minimum, and maximum values. ****P < 0.0001 (two-tailed *t* test). (**b**) Kaplan–Meier overall survival curves of patients with clear cell RCC (n = 526) according to differential expression (median) of PD-L1 or CD47. Analysis was performed with TCGA data. *P* values were calculated by log-rank test. (**c**) A comparison of PD-L1 and CD47 mRNA expression levels in human RCC cell lines (n = 31) using CCLE data. The box and whisker plot shows the median, minimum, and maximum values. ****P < 0.0001 (two-tailed *t* test). (**d**) A comparison of surface PD-L1 and CD47 expression levels in human renal cell carcinoma cell lines (n = 8) analyzed by flow cytometry. Box and whisker plot (left) shows the mean, minimum, and maximum values in all RCC cell lines. Bar graph (right) represents Δ geometric mean fluorescence intensity (MFI) ± SD of each RCC cell line averaged from three independent experiments. The Δ geometric MFI was calculated by subtracting the MFI of stained cells with isotype control from the MFI of cells stained with the antibody. (**e**) Representative histogram indicating ‘**d’** shows isotype control (gray-filled) and staining (blue-filled) with anti-PD-L1 antibody (left) or anti-CD47 antibody (right).

To evaluate whether CD47 could be a potential therapeutic target for patients with RCC, we evaluated mRNA expression levels of *CD47* and *PD-L1* and their prognostic value in RCC using TCGA datasets. The transcriptional level of *CD47* was higher than that of *PD-L1* (Fig. 1a). The *CD47* mRNA expression level was the highest in clear cell RCC, which had a worse prognosis than other histologic subtypes of RCC (i.e., papillary RCC, chromophobe RCC; Supplementary Fig. 1). High *CD47* expression levels showed a correlation with poor overall survival, whereas *PD-L1* expression did not (Fig. 1b). The same patterns in overall survival rates were consistently observed in *SIRPα* and *PD-1*, whose ligands are *CD47* and *PD-L1*, respectively. But the opposite result was shown in *THBS1* which is encoding TPS-1 (Supplementary Fig. 2). We then used the CCLE datasets to examine expression patterns of *CD47* and *PD-L1* in human RCC cell lines. Consistent with the results of TCGA data analysis, high *CD47* expression compared to *PD-L1* was detected in human RCC cell lines (Fig. 1c). Based on these transcriptional profiles from public databases, we assessed the surface expression levels of CD47 and PD-L1 in several human RCC cell lines using flow cytometry. Similar to the results from TCGA and CCLE datasets, all RCC cell lines expressed CD47 but not PD-L1 except for SNU1272 (Fig. 1d,e). CD47 was expressed at varying levels in different RCC cell lines.

### CD47 blockade increases tumor cell elimination by macrophage trogocytosis

**Figure 2:**
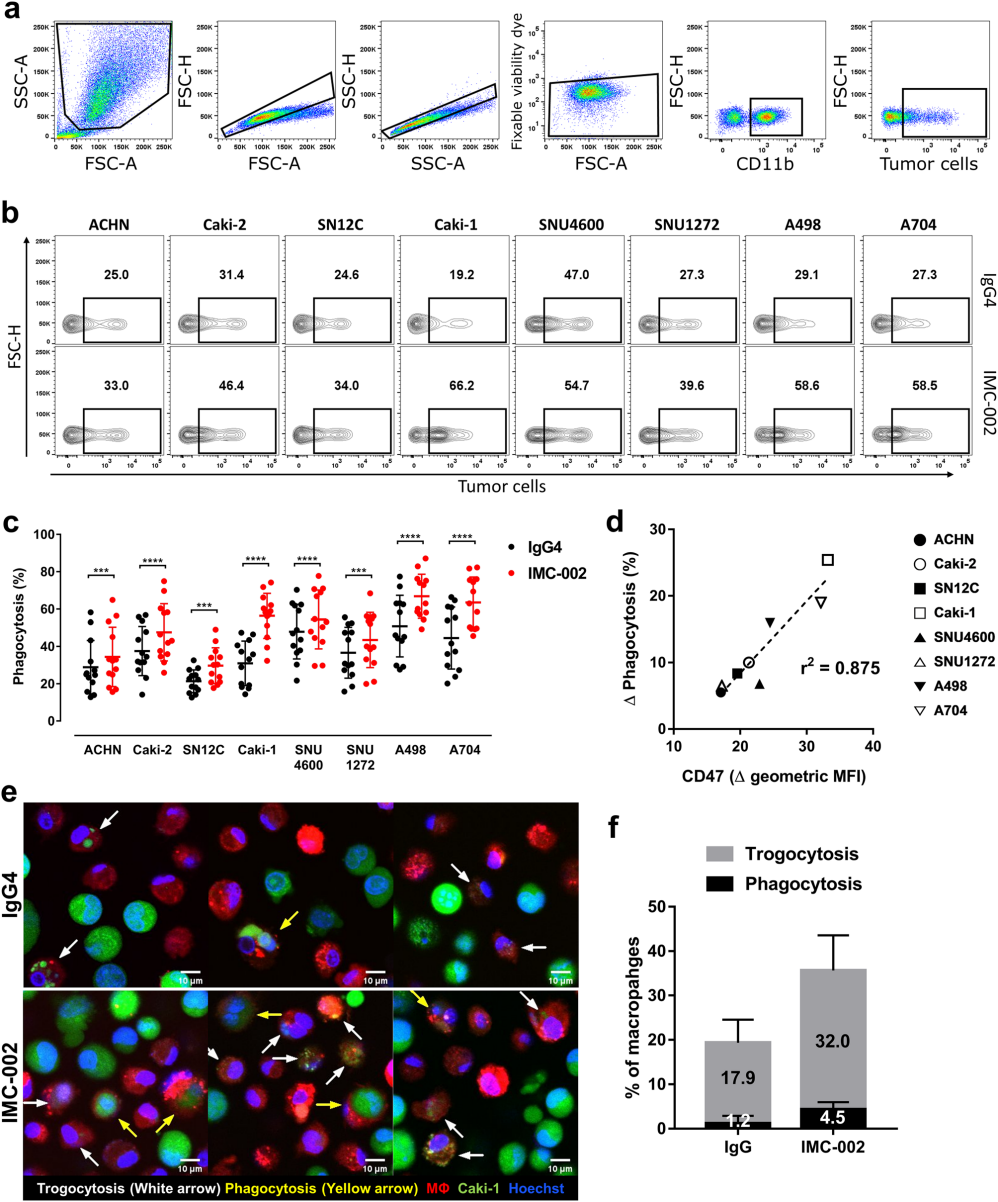
Macrophage trogocytosis is increased against RCC cell lines through CD47 blockade. (**a**) The gating strategy of flow cytometry for analyzing trogocytosis. Macrophages were discriminated staining with anti-CD11b antibody, and tumor cells were labeled with eF670 dye. Following the exclusion of doublets and dead cells, phagocytosis were assessed as the frequency of eF670^+^ events in the CD11b^+^ cell population. (**b**) Representative flow cytometry contour plot exhibiting the phagocytosis (%) of macrophages against various human RCC lines pretreated with IgG4 isotype control antibody or CD47-blocking antibody (IMC-002). (**c**) Dot and whisker plot showing phagocytosis (%) of macrophages derived from healthy donor PBMCs (n = 13). Each phagocytosis of macrophages is marked by separate dots for donors, and the mean ± SD is plotted on the graph. ***P < 0.001; ****P < 0.0001 (two-tailed *t* test). (**d**) Graph representing the relevance between CD47 surface expression levels (x-axis) and Δ trogocytosis caused by CD47 blockade (y-axis). Trogocytosis was measured by flow cytometry after coculture of tumor cells and macrophages for 2 h at an E:T ratio of 1:1. (**e**) Representative confocal images of phagocytosis assay in which Caki-1 cells (green) as target cells were cocultured with macrophages (red) in the presence of IgG4 isotype control or CD47-blocking antibody (IMC-002). (**f**) Bar graph representing the quantified level of trogocytosis or phagocytosis (%) observed by a confocal microscope.

Next, we evaluated whether interference with the CD47-SIRPα interaction could increase the phagocytic activity of macrophages against RCC cells using the CD47-blocking antibody IMC-002 (Fig. 2a). The phagocytosis assay was performed in vitro using monocyte-derived macrophages from human peripheral blood and analyzed using flow cytometry. An increase in phagocytosis caused by CD47 blockade was observed in all RCC cell lines expressing CD47 (Fig. 2b,c). High CD47 expression in RCC cells was correlated with an increase in phagocytosis due to IMC-002 (Fig. 2d). However, unlike CD47, no increase in phagocytosis was observed when PD-L1 was blocked with an anti-human PD-L1 antibody (Supplementary Fig. 3).

To understand the mechanism by which CD47 blockade affects phagocytosis by macrophages, we visualized phagocytosis and cell-to-cell interactions between macrophages and RCC cells using a confocal microscope. Unexpectedly, trogocytosis, in which macrophages “bite off” a part of a cell, occurred more frequently than phagocytosis, in which macrophages eat whole cells (Fig. 2e). This was observed even when the target cells were not opsonized by antibodies, that is, in the presence of the isotype control (IgG4). As a result of CD47 blockade, we detected a substantial increase in trogocytosis but only a marginal increase in phagocytosis (Fig. 2e,f).

**Figure 3:**
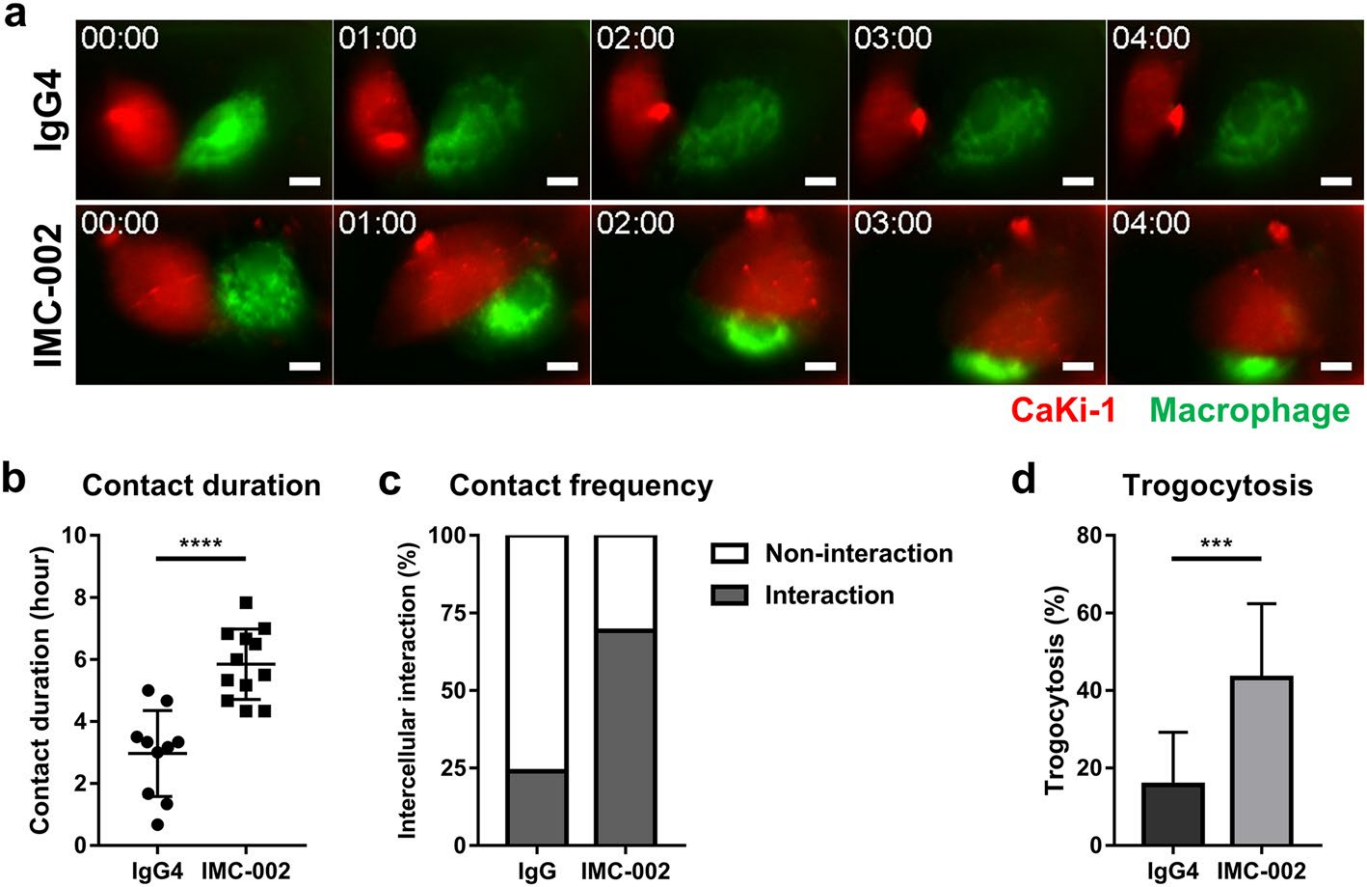
The interaction between macrophages and RCC cells is enhanced by CD47 blockade. (**a**) Snapshot of live-imaging videos (Supplementary Video 1). The Caki-1 cells (red) and macrophages (blue) were cocultured as a single cell in a microwell plate for 4 h. Each picture was taken at 1-h intervals from the start of the recording. Time displayed in the upper left of the images indicates the hour and the scale bar at the lower right represents 10 μm. (**b**) Contact duration time and (**c**) interaction frequencies of macrophages and Caki-1 cells quantified in live-imaging data. (**d**) Bar graph representing the quantified level of trogocytosis observed in the microwell plate.

In addition, visualization at the single-cell level confirmed that contact between macrophages and RCC cells was increased by blocking CD47 with IMC-002 (Supplementary Video 1; Fig. 3a). The frequency and duration of contact between these two cells increased significantly (Fig. 3b,c). An increase in trogocytosis under the CD47-blocking condition was observed, consistent with other in vitro assays presented (Fig. 3d). These data demonstrate that macrophages eliminate RCC cells through trogocytosis, which is augmented by sustained intercellular interactions after CD47 blockade.

### Combination of VEGFR TKI and CD47 blockade synergistically enhances the susceptibility of RCC cell lines to macrophages

**Figure 4:**
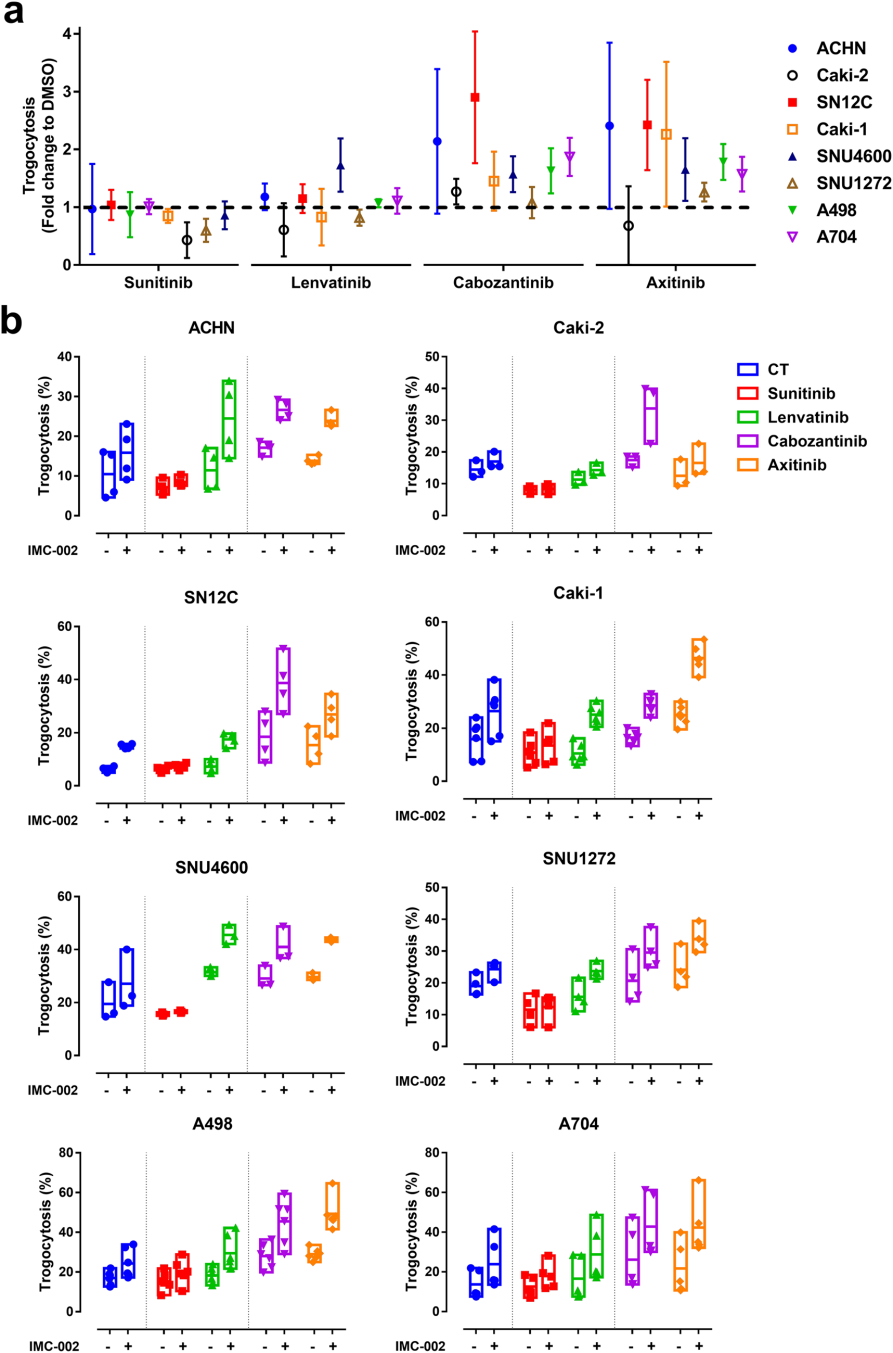
VEGFR TKIs affect the susceptibilities of RCC cell lines to macrophages. (**a**) Graph summarizing the fold change of altered trogocytosis by each VEGFR TKI in all RCC cell lines for each drug (n = 4). All RCC cells were exposed to each VEGFR TKI at a concentration of 10 μM for 24 h before the phagocytosis assay. (**b**) Trogocytosis (%) analysis of macrophages cocultured with each RCC cell line exposed to each VEGFR TKI for 24 h in the presence of IgG4 isotype control or IMC-002 (n = 3 ~ 5). Phagocytosis assay was analyzed using flow cytometry after coculture of tumor cells and macrophages for 2 h at an E:T ratio of 1:1. Dimethyl sulfoxide solution was used as a control.

We attempted to find drugs that could synergistically promote the antitumor effects of macrophages after CD47 blockade. We screened the direct cytotoxic effects of drugs on tumor cells and their effects on trogocytosis using four VEGFR TKIs that have been approved by the FDA for RCC treatment^6^. Although the direct cytotoxic effects of all VEGFR TKIs on tumor cells were low (Supplementary Fig. 4), some of the VEGFR TKIs altered the susceptibility of RCC cells to macrophages. Sunitinib (average 0.83-fold) and lenvatinib (average 1.06-fold) suppressed or did not affect trogocytosis in most RCC cell lines, except SNU4600 (Lenvatinib, average 1.73-fold). By contrast, cabozantinib (average 1.74-fold) and axitinib (average 1.67-fold) elevated trogocytosis in most RCC cell lines except SNU-1272 (average 1.08-fold, 1.26-fold; Fig. 4a). In Caki-2, trogocytosis was not increased by any VEGFR TKIs (Fold change, 0.43–1.27). To elucidate the effects of VEGFR TKIs on susceptibility to macrophages in RCC cells, we screened several molecules that act on phagocytic signaling, phosphatidylserine, calreticulin, and CD47. However, no significant differences were found (Supplementary Fig. 5).

We then assessed whether the VEGFR TKIs synergistically boosted CD47 blockade-induced trogocytosis (Fig. 4b). Cabozantinib (average 1.68-fold) and axitinib (average 1.65-fold), which increased trogocytosis as monotherapy, further elevated trogocytosis when used in combination with anti-CD47 blocking antibody in most RCC cell lines. Lenvatinib (average 1.81-fold), which did not affect trogocytosis as a monotherapy, enhanced trogocytosis when used in combination with anti-CD47 blocking antibody in most RCC cell lines except for Caki-1. Notably, the combined treatment of cabozantinib and anti-CD47 blocking antibody improved trogocytosis against Caki-2 cells, which was not affected by monotherapy (average fold change, CD47 blockade, 1.18-fold; cabozantinib, 1.21-fold; combination; 1.93-fold). However, sunitinib did not increase macrophage susceptibilities of RCC cell lines under any conditions and even abolished the trogocytic effects of CD47 blockade.

### CD11b contributes CD47 blockade-induced trogocytosis

**Figure 5:**
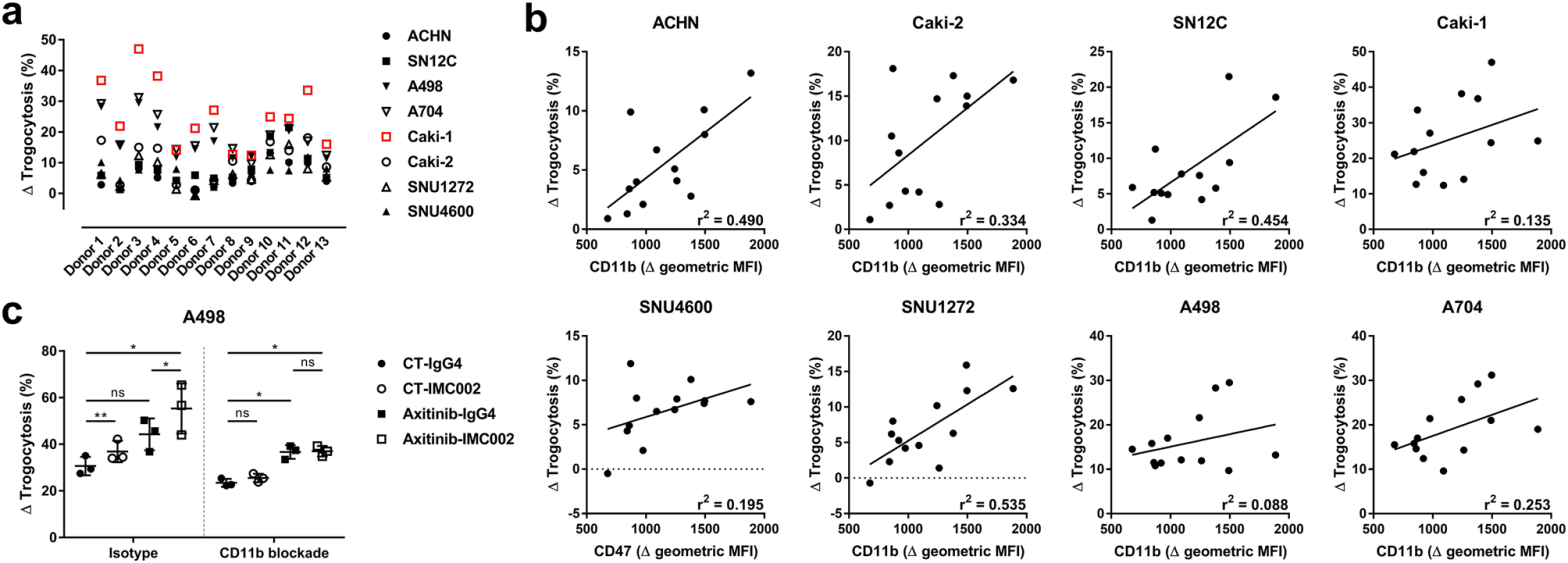
The relevance between CD11b expression level and increased levels of trogocytosis induced by CD47 blockade. (**a**) Graph showing the CD47 blockade-induced trogocytosis (%) organized by each donor. (**b**) Graphs representing the correlation between the CD11b surface expression in macrophages (x-axis) and Δ trogocytosis due to CD47 blockade (y-axis) in each RCC cell line. (**c**) Dot and whisker plot showing trogocytosis (%) of macrophages against A498 cells without and with CD11b blocking (n = 3). Each phagocytosis of macrophages is marked by separate dots for donors, and the means ± SD is plotted on the graph. *P < 0.05; **P < 0.005 (two-tailed *t* test).

We observed variation in the increased level of trogocytosis by CD47 blockade according to the donors of the macrophages (Fig. 5a). To identify the factor causing this variation, we revisited the trogocytosis assay data and determined the relevance between the altered level of trogocytosis and several factors in the macrophages. As a result, we found a correlation between CD11b expression and trogocytosis induced by CD47 blockade. High CD11b expression in macrophages resulted in a greater increase in CD47 blocking–induced trogocytosis (Fig. 5b). This was reaffirmed by confirming that the trogocytosis increased by CD47 blockade was completely abrogated by anti-CD11b blocking antibody. However, trogocytosis increased by axitinib, one of the VEGFR TKIs, was not abolished (Fig. 5c). Collectively, these results imply that CD11b contributes CD47 blockade-induced trogocytosis of macrophages.

We next examined the correlation between altered trogocytosis by CD47 blockade and SIRPα, a receptor of CD47. Unlike CD11b, the expression level of SIRPα and the increased level of trogocytosis induced by CD47 blockade were not related (Supplementary Fig. 6). Macrophages used in this study were classified as variant 1 or variant 2/7 by genotyping of *SIRPA*^35,36^. We re-analyzed the trogocytosis changes induced by CD47 blockade according to the variant types of SIRPα and found no association (Supplementary Fig. 6).

## Discussion

In this study, we found the potential of CD47 as a therapeutic target in RCC. CD47 was overexpressed compared with PD-L1 and correlated with poor prognosis in patients with RCC. We demonstrated that the antitumor effect of macrophages against RCC cell lines increases when CD47 is blocked with anti-CD47 antibodies. Considering the interaction between macrophages and tumor cells, the anti-tumor effects by blocking antibody was significantly better in CD47 than in PD-L1.

Interestingly, macrophages eliminated RCC cells mainly through trogocytosis. Trogocytosis is an action in which one cell is “bitten off” and a fraction of another cell is obtained in a cell-to-cell conjugated manner^37^. This action is discriminated from phagocytosis, which is the act of engulfing the entire cell. Trogocytosis has been verified in immune cells, such as T cells, NK cells, and neutrophils^37,38^. To date, in macrophages, trogocytosis on antibody-opsonized tumor cells has been reported^39–42^. This study is the first to show that macrophages use trogocytosis against tumor cells even in the absence of antibodies.

In addition, we demonstrated the changes induced in macrophage trogocytosis against RCC cell lines treated with each of the tested VEGFR TKIs (sunitinib, lenvatinib, cabozantinib, and axitinib). Some VEGFR TKIs showed no differences in the trogocytosis level in a single treatment condition, but most achieved the favorable effect of tumor cell removal in combination with anti-CD47 antibody IMC-002. Notably, cabozantinib and axitinib increased the susceptibility of RCC cells to macrophages as monotherapy and were synergistic with IMC-002 (Supplementary Fig. 7). Lenvatinib did not change trogocytosis when used alone but enhanced trogocytosis when combined with IMC-002. However, sunitinib did not show an increase in trogocytosis and even abrogated the elevated trogocytosis by IMC-002. Taken together, these results suggest that CD47 blockade can be an alternative or additional treatment strategy for patients who do not respond to VEGFR TKI monotherapy or ICI combination therapy; however, sunitinib may not be effective for manipulating the immune system in tumors.

We found that trogocytosis induced by CD47 blockade is abolished when CD11b signaling is blocked in macrophages. Although the detailed mechanisms were not investigated in this study, previous studies have reported that phagocytosis by macrophages is regulated through interactions between SIRPα, integrin, and Fc receptors in the phagocytic synapse^43^. The importance of CD11b/CD18 integrin in trogocytosis was also demonstrated in neutrophils^44,45^. As in the previous studies, we consistently verified that CD11b integrin is involved in trogocytosis and CD47-SIRPα signaling, suggesting that it could be predictive marker for CD47-blocking therapy.

One limitation of our study is that we did not determine which VEGFR TKI-induced changes affected the susceptibilities of RCC cells to macrophages. To determine this, further studies are needed. In addition, given that VEGFR TKIs may affect other cells in the TME, additional preclinical studies are necessary for definitive confirmation of the therapeutic effect. PD-L1 is expressed not only in tumor cells but also in various immune cells, and mainly acts to suppress immune cells in TME such as T cells. Therefore, in order to proper comparison of the therapeutic effects of PD-L1 and CD47 blockade, further in-depth study considering multiple factors is needed.

In conclusion, our study reveals that CD47 is a promising therapeutic target in patients with RCC and suggests that combination treatments of VEGFR TKIs and CD47 blockade are effective in RCC. Further investigations are still needed to understand how CD47 blockade and VEGFR TKIs synergistically improve the antitumor effect of macrophages. Nevertheless, this study suggests the potential role of CD47 and therapeutic strategy using macrophages in RCC.

## Methods

### Analysis of public data sets

The Cancer Genome Atlas (TCGA) kidney cancer RNA sequencing datasets were obtained from UCSC Xena (https://xenabrowser.net)^46^. Fragments per kilobase of transcript per million (FPKM) data were downloaded from the KIRC (n = 607), KIRP (n = 321), and KICH (n = 89) datasets, converted to log_2_(FPKM + 1), and analyzed. To analyze the correlation between overall survival and gene expression level, patients with RCC were assigned to either gene-high or gene-low groups based on the median value of gene expression. Additionally, survival analysis was performed with the survival data of each patient. The kidney cancer cell line RNA sequencing datasets were obtained from the Cancer Cell Line Encyclopedia (CCLE)^47^. Transcripts per million (TPM) data obtained from CCLE were converted to log_2_(TPM + 1) and analyzed.

### Cell lines

The human RCC cell lines used in this study, ACHN, A498, A704, Caki-1, Caki-2, SN12C, SNU-1272, and SNU4600, were purchased from the Korean Cell Line Bank (Seoul, Korea). ACHN, A498, A704, Caki-1, Caki-2, and SN12C cell lines were cultured in Dulbecco’s modified Eagle medium supplemented with 10% heat-inactivated fetal bovine serum (FBS; Gibco, Waltham, MA, USA) and 10 µg/ml gentamicin (Gibco). SNU-1272 and SNU-4600 cell lines were cultured in RPMI 1640 medium supplemented with 10% heat-inactivated FBS and 10 µg/ml gentamicin.

### Reagents and blocking antibodies

The VEGFR TKIs used in this study (sunitinib malate, cabozantinib, lenvatinib, and axitinib) were purchased from Selleck Chemicals (Boston, MA, USA). IMC-002, an IgG4 monoclonal antibody targeting human CD47, was kindly provided by ImmuneOncia Therapeutics Inc. (Yongin, Korea). Anti-human CD11b blocking antibody, IgG4 isotype control antibody, and IgG2b isotype control antibody were purchased from BioLegend (San Diego, CA, USA).

### Flow cytometry

Cell surface molecules on tumor cell lines and human macrophages were measured by flow cytometry using the following fluorochrome-conjugated antibodies: CD47-PE, CD47-APC, PD-L1-PE, and CD11b-PE (BD Biosciences, Franklin Lakes, NJ, USA). CD172a-PerCP-eF710 was purchased from eBioscience (Thermo Fisher Scientific, Waltham, MA). Flow cytometry analysis was performed using a FACS Calibur or FACS Canto II (BD Biosciences), and the data were analyzed using FlowJo v10.7 software (FlowJo LLC, Ashland, OR, USA, https://www.flowjo.com/).

### Macrophages generation

Peripheral blood mononuclear cells (PBMCs) were isolated by Ficoll density gradient separation from healthy donor peripheral blood collected using a leukocyte reduction system chamber. Monocytes were isolated from PBMCs by negative selection using a pan monocyte isolation kit (Miltenyi Biotec, Bergisch Gladbach, Germany) according to the manufacturer’s recommendations. Freshly isolated monocytes were cultured in RPMI 1640 medium supplemented with 10% heat-inactivated FBS, 1% penicillin/streptomycin, 2 mmol/L L-glutamine (Gibco), and 25 ng/ml recombinant human macrophage colony-stimulating factor (Gibco). The medium and cytokines were replaced every 2–3 days, and differentiated macrophages were harvested on day 7 to be used for the functional assay.

### Phagocytosis assay using flow cytometry

Target cells were labeled with cell proliferation dye eFlour 670 (Invitrogen, Carlsbad, CA, USA) or CellTrace CFSE (Invitrogen) and preincubated with 5 µg/ml IMC-002 or Ultra-LEAF purified human IgG4 isotype control recombinant antibody (BioLegend), respectively. For an E:T ratio of 1:1, 5 × 10^4^ target cells and human macrophages were cocultured in a u-bottom 96-well plate. After 2 h of incubation, whole cells were collected in round-bottom test tubes and stained with CD11b-PE (BD Biosciences) or CD11b-APC (BD Biosciences). The stained samples were analyzed using a FACS Canto II (BD Biosciences) on the same day after staining. Then, the data were analyzed using FlowJo v10.7 software (FlowJo LLC). To confirm the effect of VEGFR TKIs on phagocytosis, target cells were plated at 2 × 10^5^ cells/well in 6-well plates. After allowing the cells to adhere overnight (16–18 h), they were treated with VEGFR TKI (10 µM) for 24 h. Thereafter, the phagocytosis assay proceeded in the same manner as described above.

### Phagocytosis assay using confocal microscopy

The cells were labeled with 1 µM CellTrace Far Red or CellTrace CFSE at 37 °C for 10 min. After washing once with complete media, the cells were labeled with 3 µM Hoechst to stain the nuclei at 37 °C for 15 min. For an E:T ratio of 1:1, 5 × 10^5^ labeled macrophages and CaKi-1 cells were cocultured in FACS tubes. After 4 h of incubation, the cells were collected and placed in a confocal dish for imaging. A confocal laser scanning microscope (FV 1200, Olympus) with a 40 × objective lens (UPLSAPO 40 × 2; Numerical Aperture = 0.95) was used. The acquired images were processed and analyzed using ImageJ (https://imagej.nih.gov/ij/).

### Live cell imaging in microwell

A modified Olympus IX83 epifluorescence microscope with a 40× objective lens (UPlanFLN; NA = 1.30) and an ANDOR Zyla 4.2 sCOMS camera were used for imaging experiments. For fluorescence imaging, a U-LH75XEAPO Xenon lamp (75 W, Olympus) and yellow (EX BP 530/30, BS 550, EM BP 575/40) and Cy5 (EX BP 620/60, BS 660, EM BP 770/75) filters were used. The microscope was automatically controlled by Micro-Manager, and the acquired images were analyzed and processed with ImageJ.

To observe an interaction between a macrophage and cancer cell, microwell arrays were fabricated using double replicas of a silicon master fabricated by standard photolithography. In addition, 85 µm × 50 µm square microwell arrays were fabricated on a silicon wafer with negative photoresist SU-8 50 and developer. The silicon master was treated with trichloro(1H,1H,2H,2H-perfluorooctyl) silane. Then, we poured the polydimethylsiloxane precursor mixture (Sylgard 184; base:curing agent = 10:1) on the silicone master and cured it at 70 °C for 4 h to replicate. The replicated polydimethylsiloxane mold was placed on a glass surface functionalized with an acrylate group, perfused with the precursor solution poly(ethylene glycol) dimethacrylate, and cured with a UV light. Hydrogel microwells were treated with air plasma (100 W) for 1 min and coated with 2 µM fibronectin solution at 37 °C for an hour. Macrophages and cancer cells were labeled with 1 µM CellTrace Far Red or CellTrace Yellow at 37 °C for 10 min, respectively. These labeled cells were loaded on the fibronectin-coated microwell array (1 × 10^5^ cells/ml). Time-lapse imaging was started 1 h from cell addition to wait for the cells to spread to the surface.

### Cell viability assay

Cell viability assays were performed as in the previous study^48^. The RCC cell lines were plated at 3,000 cells per well in 96-well plates. The cells were exposed to various VEGFR TKIs (sunitinib, cabozantinib, lenvatinib, and axitinib) for 72 h in eight conditions from 0 to 10 μM in 1/2 serial dilutions. Dimethyl sulfoxide solution (Gibco) was used as a control. Cell viability was analyzed using a CellTiter-Glo Luminescent cell viability assay kit (Promega, Madison, WI, USA). The luminescent signal was measured using a GloMax Discover microplate reader (Promega).

### Genotyping of *SIRPA* variants

Genomic DNA was extracted from healthy donor PBMCs using a GeneAll Exgene Cell SV kit (GeneAll Biotechnology, Seoul, Korea). Exon 3 of *SIRPA* was amplified using EconoTaq PLUS GREEN 2X master mix (Lucigen Corporation, Middleton, WI, USA). The PCR thermal cycle was conducted on a Veriti 96-well thermal cycler (Applied Biosystems, Waltham, MA, USA) as follows: initial denaturation at 94 °C for 2 min; 35 cycles of 94 °C for 30 s, 58 °C for 30 s, and 72 °C for 1 min 40 s; final extension at 72 °C for 7 min. Amplicons were purified using Wizard SV gel and a PCR clean-up system (Promega), and the sequences were confirmed by Sanger sequencing. The primers used for PCR amplification and Sanger sequencing are as follows: PCR, (forward) 5′-AAACACACTGGCACGAGTCTA-3′, (reverse) 5′-GCCTTAACGGAGGAACCCAA-3′; Sanger sequencing, (forward) 5′-AGAATACAGGCTCATGTTGCAGGT-3′, (reverse) 5′-GCCTTCAGCAAATAGCATGACGT-3′.

### Statistical analysis

Statistical significance was analyzed by GraphPad Prism v.7.0 (GraphPad Software, San Diego, CA, https://www.graphpad.com/). Data are presented as the means ± SD. Student two-tailed *t* test were used to compare two groups. *P* values < 0.05 were considered significant. *P < 0.05; **P < 0.005; ***P < 0.001; ****P < 0.0001.

## Supplementary Information

Supplementary Information 1.

Supplementary Video 1.

## Data Availability

As mentioned in the Methods, the main datasets used and analyzed in this study are available in the USCS Xena, (https://xenabrowser.net/datapages/) GDC TCGA Kidney cancer datasets. Gene expression data of cell lines (CCLE dataset) were obtained from the depmap portal, (https://sites.broadinstitute.org/ccle/datasets) DepMap Public 22Q2 Primary Files, CCLE_expression.csv. All other data used in this study are available from the corresponding authors for reasonable purposes.
