# Supplementary material for: Blockade of CD47 enhances the antitumor effect of macrophages in renal cell carcinoma through trogocytosis
Source: Sci Rep. 2022 Jul 22;12:12546. doi: 10.1038/s41598-022-16766-3 (PMC9307775; doi:10.1038/s41598-022-16766-3)
Supplement: Supplementary file 1 — Supplementary Information 1. [file 41598_2022_16766_MOESM1_ESM.docx]

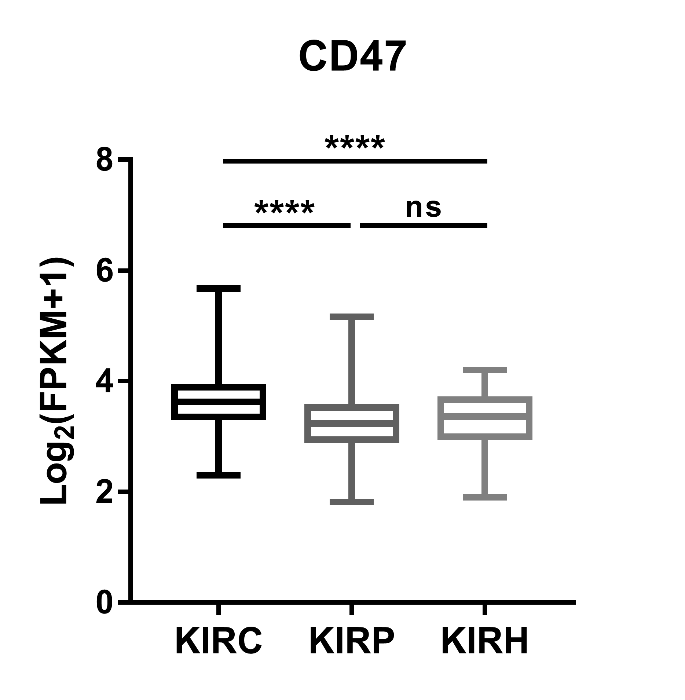


Supplementary Figure 1. High *CD47* transcript levels in patients with clear cell RCC.

The mRNA expression level of *CD47* for each type of RCC was analyzed using TCGA data obtained from UCSC Xenabrowser. *****P* < 0.0001 (two-tailed t-test).


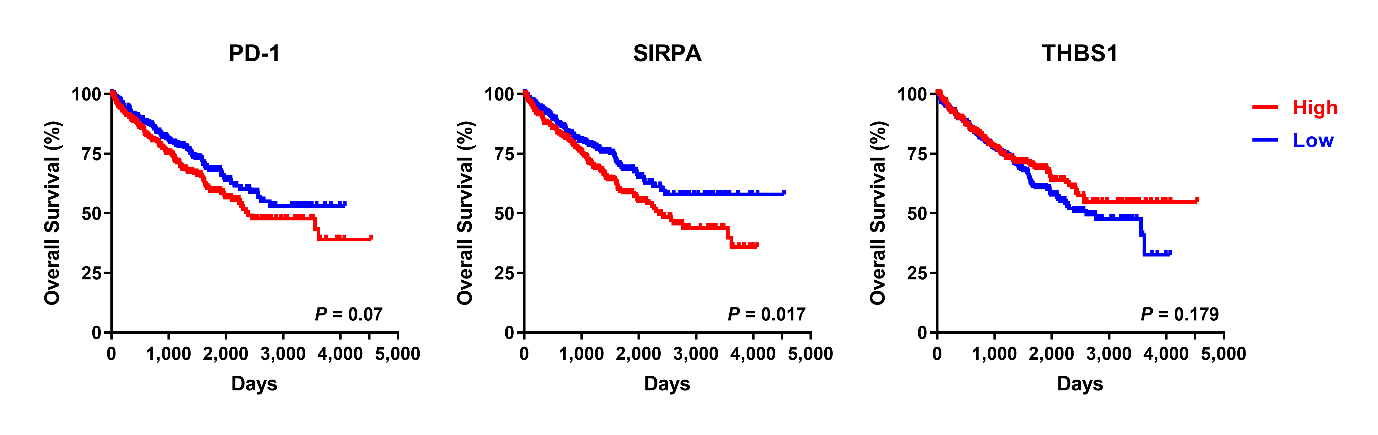


Supplementary Figure 2. Overall survival rate of clear cell RCC patients according to *PD-1* or *SIRPA* expression level.

Kaplan-Meier overall survival curves of patients with clear cell RCC (n = 526) according to differential expression levels of *PD-1, SIRPA,* or *THBS1* (median). Analysis was performed using TCGA data obtained from UCSC Xenabrowser. *THBS1* is the gene encoding TSP-1.


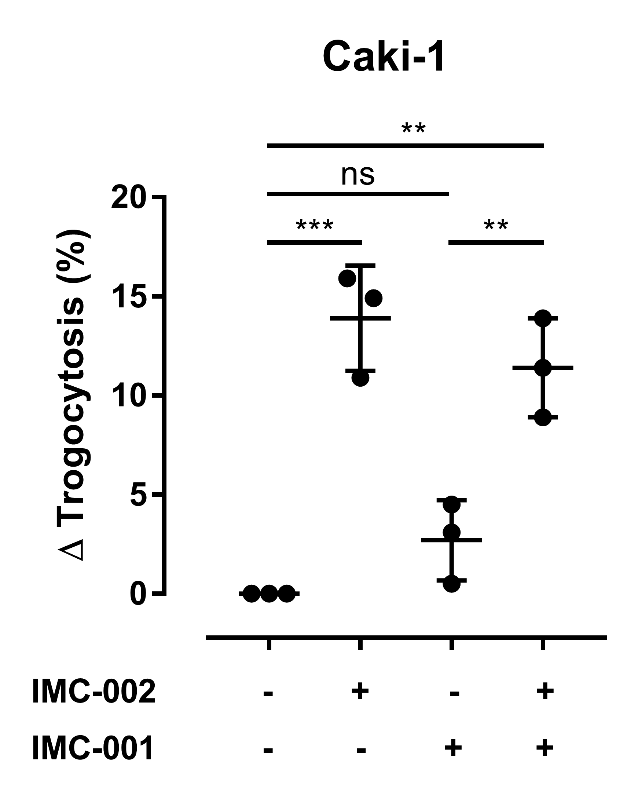


Supplementary Figure 3. Trogocytosis by macrophages of Caki-1 cells (CD47^+^/PD-L1^−^) is enhanced by the anti-CD47 antibody but not by the anti-PD-L1 antibody.

Caki-1 cells were pretreated with each antibody (10 μg/ml) for 30 minutes, then cocultured with macrophages at 1:1 ratio for 2 hours (n = 3). Δ Trogocytosis (%) = trogocytosis in with anti-CD47 antibody (IMC-002) or anti-PD-L1 antibody (IMC-001) and – trogocytosis with isotype controls (IgG4 or IgG1). ***P* < 0.005; ****P* < 0.001 (two-tailed t-test); ns, not significant.


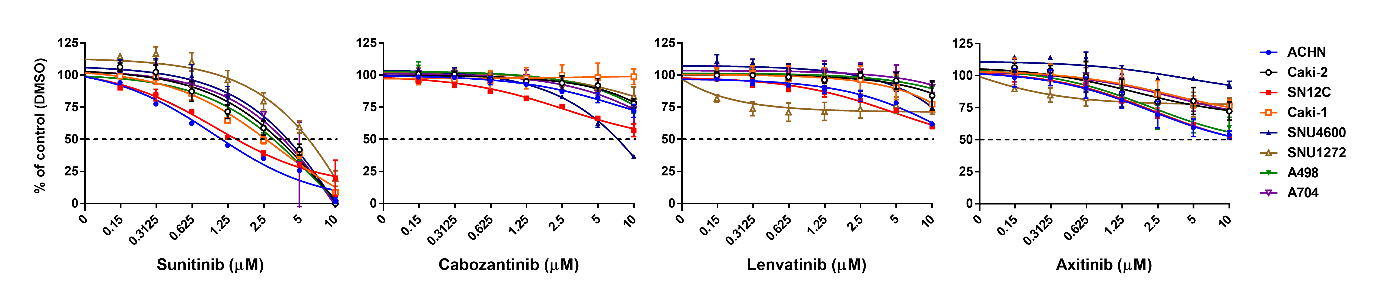


Supplementary Figure 4. Direct cytotoxic effects of VEGFR TKIs against RCC cell lines.

Each RCC cell line was treated with each VEGFR TKI (sunitinib, cabozantinib, lenvatinib, and axitinib) for 72 hours in eight conditions from 0 to 10 µM in ½ serial dilutions. Dimethyl sulfoxide solution was used as a control.


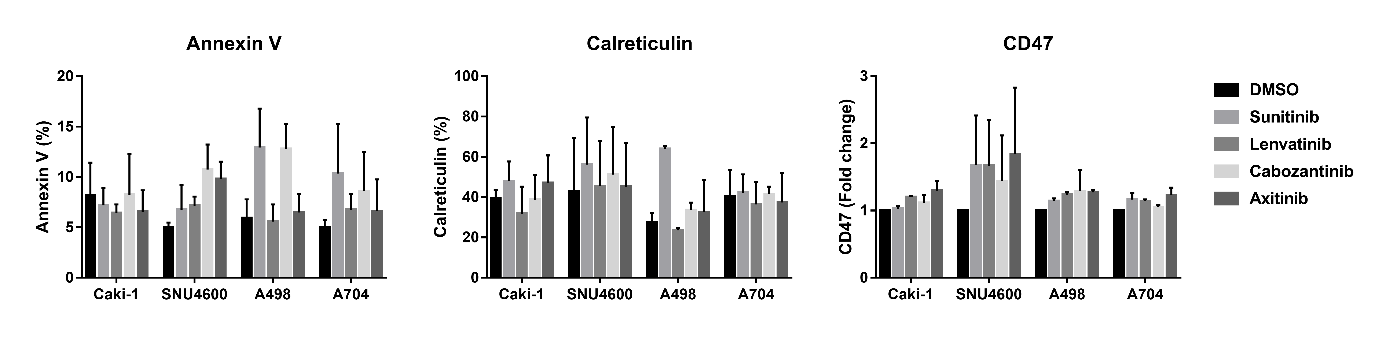


Supplementary Figure 5. Changes in expression of surface molecules in RCC cell lines after VEGFR TKIs exposure.

Bar graphs represent the expression level of phosphatidylserine (Annexin V), calreticulin, or CD47 in several RCC cell lines (Caki-1, SNU4600, A498, and A704) measured using flow cytometry. These molecules were assessed in RCC cells exposed to each VEGFR TKIs (sunitinib, lenvatinib, cabozantinib, and axitinib) at a concentration of 10 μM for 24 hours. Dimethyl sulfoxide solution was used as a control. Annexin V and calreticulin were analyzed as a percentage (%) of the positive cell population among the living cells. CD47 expression data are presented as fold changes compared with the control. All experiments were independently performed three times and indicated data as the means ± SD.


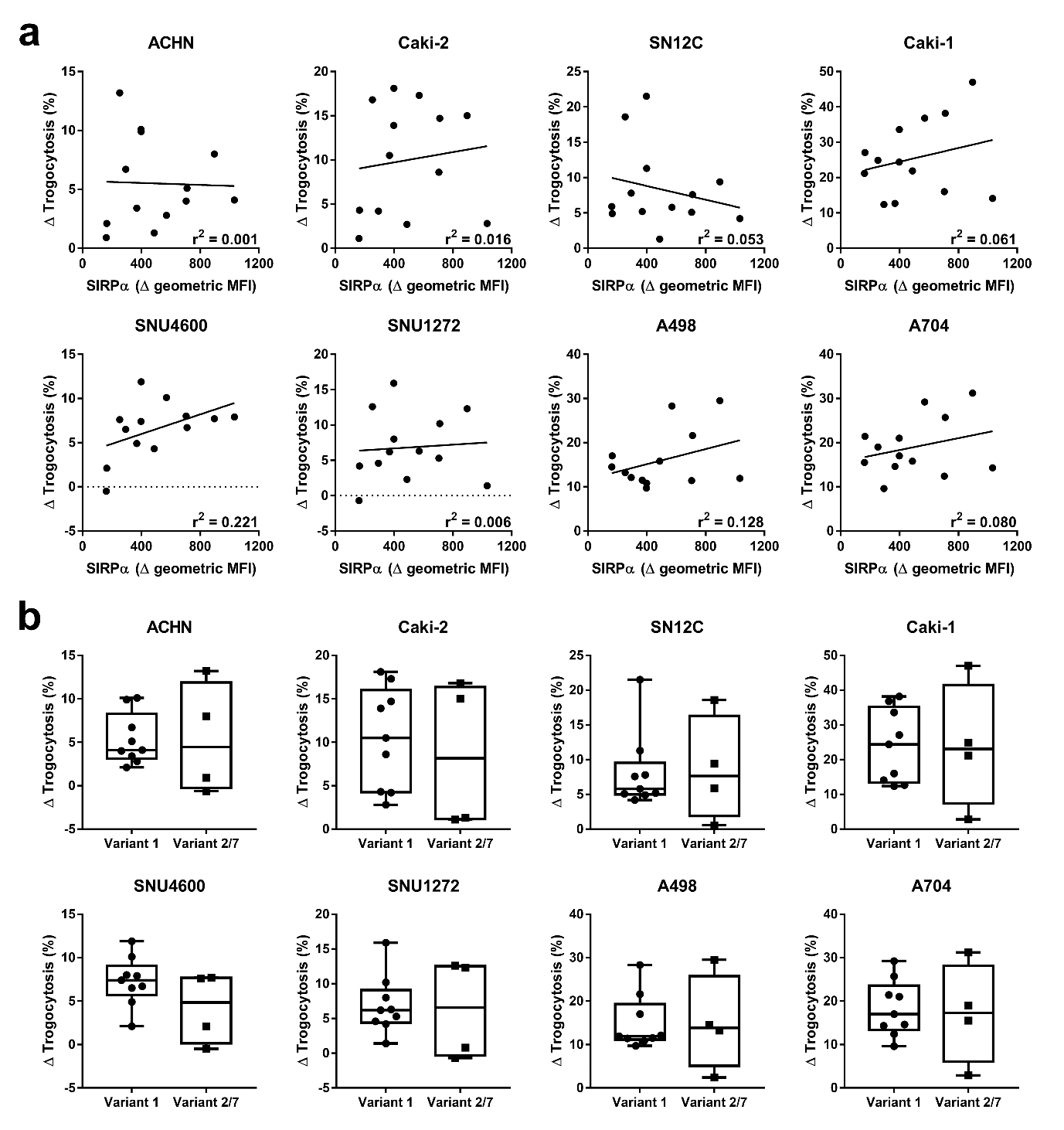


Supplementary Figure 6. SIRPα variants and expression levels were not associated with increased levels of trogocytosis induced by blocking CD47.

**a** Each graph represents the correlation between the SIRPα surface expression in macrophages (x-axis) and Δ trogocytosis due to CD47 blockade (y-axis) in each RCC cell line. **b** Box and whisker plots show the results of analyzing the phagocytosis data in Fig. 2C by revisiting and classifying it according to SIRPα variants.


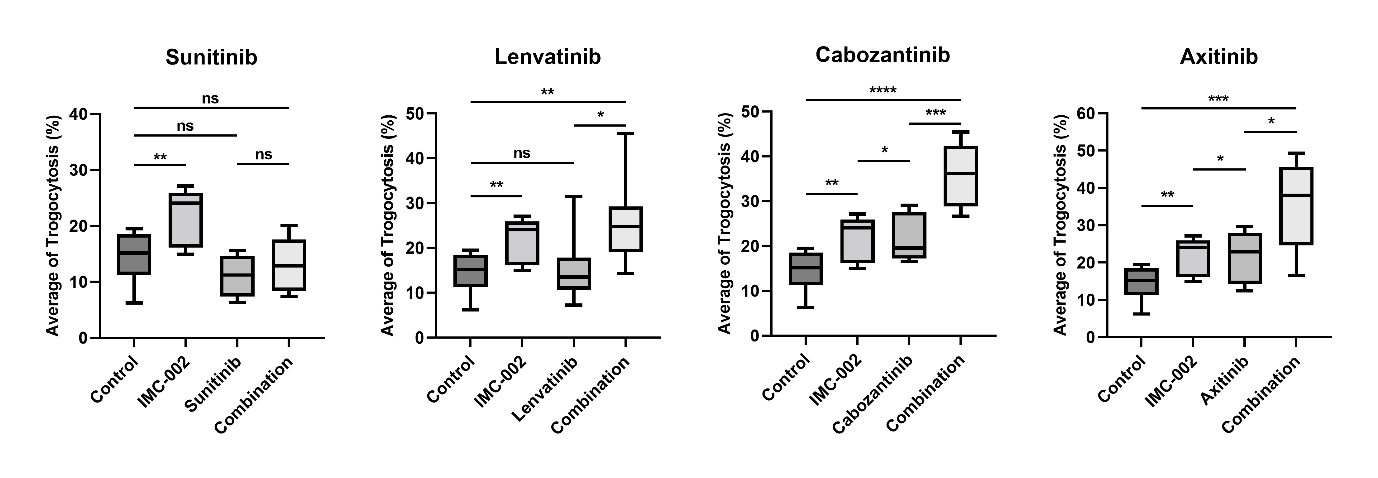


Supplementary Figure 7. Changes in trogocytosis under different treatment conditions.

Box and whisker plots show the results of analyzing the phagocytosis data in Fig. 4 by revisiting and analyzing it according to each VEGFR TKIs treatment condition. Trogocytosis levels (%) in each RCC cell line were averaged and displayed in the graph.

**Supplementary Video 1. Live cell imaging of interaction between macrophages and Caki-1 cells.**

Macrophages (Green) and Caki-1 cells (Red) were co-cultured in a micro-well plate for 4 hours with **a** IgG4 isotype control or **b** IMC-002. Cells were plated in 1-3 cells in each well. Videos were recorded an hour after co-culture using a confocal microscope.
